# The combination of LILRB4-targeting NK cell engagers and cGAS–STING agonists enhances the anti–multiple myeloma immune activity of NK cells

**DOI:** 10.1371/journal.pone.0339375

**Published:** 2025-12-19

**Authors:** Meng Li, Yuwei Zhao, Lizhou Zhao, Xue Chen, Jianxun Kang, Aiping Tong, Chen Yang, Ping Wang, Minjun Yang, Kejiang Wang, Jialiang Gao, Ying Li, Xuemei Fu

**Affiliations:** 1 Blood Research Laboratory, Chengdu Blood Center, Chengdu, Sichuan Province, China; 2 Department of Biotherapy, State Key Laboratory of Biotherapy and Cancer Center, Research Unit of Gene and Immunotherapy, Chinese Academy of Medical Sciences, Collaborative Innovation Center of Biotherapy, West China Hospital, Sichuan University, Chengdu, Sichuan Province, China; 3 The Department of Experimental Medicine, Meishan City People’s Hospital, Meishan, Sichuan Province, China; Rutgers: Rutgers The State University of New Jersey, UNITED STATES OF AMERICA

## Abstract

Although significant progress has been made in immune-targeted therapy for multiple myeloma (MM), it remains highly recurrent and incurable. Consequently, there is still an urgent need to develop more effective strategies against the recurrent and refractory tumor subsets. LILRB4, which is expressed on MM and Myeloid-Derived Suppressor Cells (MDSC) and plays an important role in promoting tumor progression and regulating the immuno-suppressive microenvironment. Therefore, it is an attractive target for MM treatment. We developed a bispecific natural killer cell engager (BiKE), LILRB4/CD16A, to mediate NK cell-mediated lysis of MM cells. BiKE combined with CD16A strongly activated NK cells derived from human peripheral blood, and at the same time, BiKE bridged tumor cells to NK cells and promoted NK cytotoxicity, showing significant antitumor activity against LILRB4-high MM cells. NK cell cytotoxicity was further enhanced by the combined stimulation of innate immunity using cGAS-STING signaling agonists. In xenograft MM tumor models in immunodeficient mice, NK cells derived from human peripheral blood and expanded in vitro were combined with BiKE and cGAS-STING signaling agonists, demonstrating effective anti-tumor activity and inhibition of MM cell proliferation. Collectively, the combination of cGAS-STING agonists and LILRB4-targeting NK cell engagers offers a promising approach for treating relapsed/refractory MM.

## Introduction

Multiple myeloma (MM) is a genetically heterogenous and relapsing clonal plasma cell carcinoma. It is the second most common hematologic malignancy which accounts for 15–20% of hematologic malignancies and has a 5-year survival rate of 60% [1]. MM could affect multiple organ systems, leading to hypercalcemia, renal failure, bone destruction, blood disorders and frequent infections. Most patients ultimately relapse with resistant disease [2]. Currently, therapeutic strategies such as autologous hematopoietic stem cell transplantation, immunomodulators, and immune-targeted therapies have been employed to improve survival outcomes in MM patients. However, relapse is almost inevitable due to its high degree of heterogeneity, the presence of multiple variants, and the frequent development of drug resistance [1]. To address the growing need for more effective treatments, it is crucial to develop novel drugs that target new mechanisms or previously unexplored targets to overcome the issues of drug resistance and relapse in MM patients, thereby significantly improving patient prognosis and life quality.

Immuno-oncology has emerged as a revolutionary approach in cancer treatment, with notable advancements including antibody-drug conjugates (ADCs), CAR-T cells, and cell conjugates. CAR-T cells, engineered to recognize specific tumor antigens (TAs), have demonstrated remarkable clinical efficacy in some hematological malignancies. However, CAR-T cell therapy is frequently associated with significant adverse events, including cytokine release syndrome (CRS) and neurotoxicity [3,4]. In parallel, T cell engagers, which redirect endogenous immune effector T cells to tumor sites, also face limitations due to toxicity. Recently, interest has focused on the use of natural killer (NK)cells for therapeutic interventions, given their inherent anti-tumor properties. A CAR-NK study targeting both BCMA and GPRC5D demonstrated promising therapeutic efficacy to MM [5]. Nevertheless, the generation of CAR-NK is challenging due to the unique immunological properties of NK cells that impede efficient viral transduction. Therefore, this study aims to produce an NK cell engager and combine it with NK cell adoptive therapy for MM, which can circumvent the challenges associated with CAR-NK preparation while maintaining antigen specificity.

The CD16A receptor (FcgRIIIa) on NK cells is central to the ADCC mechanism, as it binds to the Fc region of IgG antibodies attached to the target cells. The clinical significance of CD16A interaction with antibody Fc region is demonstrated by the success of several FDA-approved cancer therapies, such as rituximab [6], trastuzumab [7], and cetuximab [8], all of which depend on CD16A receptor activation to exert their anti-tumor effects [9].Unlike the transient and relatively low-affinity interaction between the Fc domain of monoclonal antibodies (mAbs) and CD16A, Bispecific natural killer cell engager (BiKE) can establish a stable connection with any activating receptor on NK cells, which significantly enhance ADCC efficiency [10]. Anti-CD16A antibodies are attractive candidates and have been employed to build BiKE [11]. For instance, the BCMA/CD16A bispecific NK engager demonstrated a potent MM cell-killing activity [12]. Despite the effective use of NK cells, challenges such as tumor cell target loss and recurrence persist. Therefore, it is essential to explore new targets for targeted killing.

Leukocyte immunoglobulin-like receptor B4(LILRB4) is an immunosuppressive receptor for members of the LILRB family. It inhibits T-cell function and promotes leukemic cell infiltration by creating an immunosuppressive microenvironment in monocytic leukemic cells [13]. Recent studies have shown that LILRB4 is predominantly expressed in the precursor stage of plasma cells (CD38 + CD138 + CD19 + CD56+), with expression decreasing in mature malignant plasma cells (CD38 + CD138 + CD19-CD56+), although both are still higher than in normal plasma cells (CD38 + CD138 + CD19 + CD56-). Moreover, compared to newly-diagnosed MM patients, higher LILRB4 expression is observed in those who relapse after treatment, highlighting its significance in MM progression and drug resistance. Considered as a biomarker for invasive MM [14]. Conversely, LILRB4 knockdown or antibody blockade significantly inhibits MM cell proliferation both in vitro and in vivo [15,16]. Overexpression of LILRB4 in MM patients is associated with poor prognosis and decreased overall survival [17]. Furthermore, CAR-T therapy targeting LILRB4 has been shown to effectively eliminate MM cells and partially disrupts the immunosuppressive tumor microenvironment [18]. Thus, LILRB4 is a promising target for MM treatment.

The cyclic GMP-AMP synthase (cGAS)–stimulator of interferon genes (STING) pathway represents a central innate immune mechanism with potent antitumor activity. As a cytosolic DNA sensor, cGAS detects exogenous DNA and generates cGAMP, which binds and activates STING. Activated STING then engages IRF3 and NF-κB, driving the production of type I interferons and proinflammatory cytokines. This cascade amplifies cytotoxic immune responses, making cGAS–STING activation critical for promoting anticancer immunity [19]. Studies have demonstrated that cGAS-STING pathway agonists significantly enhance the anti-tumor activity of NK cells [20–23].

In our study, BiKE: LILRB4/CD16A was developed, which specifically links NK cells [24] with LILRB4-positive MM cells to facilitate tumor cell killing. To improve the efficacy of cancer immunotherapy, we employed the cGAS–STING agonist SR-717 in combination with the BiKE LILRB4/CD16A for treatment. In vitro, we demonstrate the ability of cGAS-STING signaling agonists combined with BiKE to promote effective killing of LILRB4 ^+^ MM cells by NK cells in a co-culture system. The efficacy of BiKE and SR-717 in assisting NK cells in adoptive therapy of MM was also verified in vivo, and this treatment regimen could significantly kill LILRB4 ^+^ MM cells in vivo and inhibit tumor cell growth. This approach provides a novel therapeutic strategy for NK cell-targeted therapy in relapsed/refractory MM.

## Materials and methods

### Mice

Female C-NKG mice, aged six to eight weeks, were obtained from Cyagen Biosciences Inc. (Guangzhou, China) and used after a minimum one-week acclimatization period. The animals were housed in cages with automatic water bottles, maintained on a 12-hour light/dark cycle at 22°C, and provided standard certified commercial laboratory pellets and water ad libitum. All animal care and use procedures were approved by the Ethics Committee of Chengdu Blood Center (Ethical Review (Research) No.07, 2024).

### Cell lines

MM1.S, RPMI8226, and K562 cell lines were purchased from the American Type Culture Collection (ATCC, USA). Peripheral blood mononuclear cells (PBMCs) were obtained from healthy volunteers, with all samples collected under written informed consent and approved by the Ethics Committee of the Chengdu Blood Center. All cell lines were cultured in RPMI1640 medium (Gibco, USA) supplemented with 10% fetal bovine serum, while PBMCs were cultured in RPMI1640 medium containing 10% inactivated fetal bovine serum. Cells were incubated at 37°C in a humidified incubator with 5% CO_2_.

### Human NK cell isolation and ex vivo expansion

PBMCs were isolated from human peripheral blood using density gradient centrifugation with Ficoll (DAKEWE, China) as previously described [25]. They were then purified by negative selection using CD3 magnetic beads and CD56 positive selection with magnetic beads (Miltenyi Biotec, Germany). The cultures were maintained in NK medium (Miltenyi Biotec, Germany) containing 5% human AB serum (GeminiBio, USA) and 500 IU/mL IL-2 (Miltenyi Biotec, Germany). The cells were activated using the NK cell activation/expansion kit (Miltenyi Biotec, Germany) for 6 days, followed by expansion in a 5% CO_2_, 37°C incubator with medium without activated magnetic beads. NK cells were initially seeded at a density of 1 x 10^6^/mL (range: 0.8–1.3 x 10^6^). After 6 days, the cells were inoculated into T25 flasks at a density of 1–1.5 x 10^6^/mL. Cell counts were performed every 2–3 days, and the flasks were divided and supplemented with complete medium.

### Detection of CD69 and cytotoxic function markers CD107a, IFN-γ and TNF-α

NK cell function was assessed by flow cytometry as previously described [26]. Briefly, PBMCs and target cells were co-cultured, and after 1 hour, the protein transport inhibitor Golgi Stop (BD Biosciences, USA) was added. The cells were then incubated for an additional 3 hours. Cells were stained with the Live/Dead Fixable Aqua staining kit (Thermo Fisher, USA), APC-conjugated anti-CD69 (Cat: 985206, BioLegend, USA), APC-conjugated anti-CD107a (Cat: 328620, BioLegend, USA), Pacific Blue-conjugated anti-CD56 (Cat: 985914, BioLegend, USA), and FITC-conjugated anti-CD3 (Cat: 300406, BioLegend, USA). Intracellular cytokines IFN-γ and TNF-α were stained after surface staining with CD3-FITC and CD56- Pacific Blue/CD56-APC (Cat: 985906, BioLegend, USA), followed by fixation with 4% paraformaldehyde and permeabilization with 0.1% Triton X-100. Permeabilized cells were stained with BV421-conjugated anti-IFN-γ (Cat: 506538, BioLegend, USA) and APC-conjugated anti-TNF-α (Cat: 502912, BioLegend Biosciences, USA), washed with PBS, and analyzed using Novocyte flow cytometry.

### Vector construction, expression and purification of BiKE

The amino acid sequences of anti-LILRB4 scFv and anti-CD16A scFv, including VL and VH domains, were derived from patents US20210179687 and US2021/0253698 A1, respectively. The VL and VH domains within the CD16A scFv in the pVAX vector are linked by a (Gly_2_Ser)_7_ peptide, while those within the LILRB4 scFv are linked by a (Gly_4_Ser)_3_ peptide. The CD16A scFv and LILRB4 scFv are linked by the human IgG_4_-Fc segment, resulting in the construct CD16A(scFv)-Gly_4_Ser-hFc-(Gly_4_Ser)_3_-LILRB4(scFv). Hexa-histidine (His6) tags were fused to the C-terminus of the BiKE construct.

BiKE is expressed in CHO cells [27]. Briefly, vector plasmids were transfected into CHO cells by electroporation and cultured for 10 days with shaking. The cell culture supernatant was collected and purified using high-affinity Protein A + G (Beyotime, China) to isolate the human Fc segment, followed by antibody concentration using a shutoff column.

### Antibody affinity testing

For antibody affinity testing, LILRB4 (hFc-tagged) and CD16A (6His-tagged) recombinant proteins(TargetMol, USA) were coated onto 96-well plates at 50 ng/well and incubated overnight at 4°C. 2% BSA was used to block the plates for 2 hours at 37°C, followed by the addition of gradient concentrations of antibody, which were incubated for 1.5 hours at 37°C. The plates were washed five times with PBST and patted dry, followed by the addition of goat anti-human IgG (H + L) or goat anti-6His tag secondary antibody (Proteintech, China). The plates were incubated at 37°C for 1 hour. The plates were washed five times with PBST, patted dry, and 100 μL of TMB was added for color development (Solarbio, China), followed by termination with 50 μL of concentrated sulfuric acid. Finally, absorbance at 450 nm was measured using a microplate reader.

### Western blot analysis

Total protein was extracted from cells using RIPA lysate (Beyotime, China) as previously described [28], Protein samples (20 μg) were separated by 10% SDS/PAGE and transferred to PVDF membranes (Millipore, USA). The membranes were blocked with 5% nonfat dry milk for 2 hours, incubated overnight at 4°C with primary antibodies (1:1000 dilution, rabbit anti-human, Proteintech), Rabbit anti-human LILRB4 antibody was used to detect the expression of LILRB4 in MM cell lines, which used GAPDH as an internal reference. Following primary antibody incubation, membranes were washed three times with TBST for 15 minutes each, and then incubated with goat anti-rabbit IgG (H + L) secondary antibodies (1:5000, Proteintech) for 1 hour at room temperature on a shaker. The membranes were washed three times with TBS-T for 15 minutes, developed using chemiluminescence reagent (Bio-Rad, USA), and detected with a chemiluminescence imager.

### Flow cytometry

NK cells were identified as CD56^+^CD3^-^, and their proportion and CD16 expression on NK cell membranes were assessed by direct labeling with CD56- Pacific Blue (Cat: 985914, BioLegend, USA), CD3-FITC (Cat: 300406, BioLegend, USA), and CD16-PE antibodies ((Cat: 360704, BioLegend, USA). Briefly, the cell suspension was collected, centrifuged to remove the medium, and washed once with PBS. Antibodies were incubated for 30 minutes at room temperature, washed twice with PBS, and analyzed using a Novocyte flow cytometer. The affinity of BiKE for NK cells and target cells was assessed using indirect labeling flow cytometry. Briefly, BiKE and the control monoclonal antibody (20 µg/ml) were incubated with cells for 1 hour at room temperature, washed twice with PBS, and incubated with FITC-labeled goat anti-human IgG (H + L) secondary antibody (1:1000, Cat: SA00003−12, Proteintech, China) for 20 minutes at room temperature. Additionally, the same indirect labeling method was used to detect LILRB4 expression on MM cell membranes by flow cytometry, using rabbit anti-human LILRB4 (1:1000, Cat: 28422–1-AP, Proteintech, China) as the primary antibody, followed by FITC-labeled goat anti-rabbit IgG (H + L) secondary antibody (1:1000, Cat: SA00003−1, Proteintech, China). Cells were then washed twice with PBS and analyzed using Novocyte flow cytometry.

### Assay of NK cell cytotoxicity to tumor cell lines

MM1.S cells were washed with PBS and resuspended in 2μM calcein-AM (Beyotime, China) solution at a density of 1 x 10^6^/mL. The cells were incubated at 37°C for 30 minutes, as previously described [29]. Labeled MM1.S cells were washed twice with PBS. Target cells were then seeded at a 1:1 ratio with NK cells in U-bottom 96-well plates and co-cultured for 4 hours at 37°C. The control group contained only target cells (spontaneous release), while the maximum release group was treated with 0.2% Triton X-100. After centrifugation of the cells in the 96-well plates, 100 μL of supernatant was transferred to black-walled 96-well plates (Corning, Costar). Fluorescence signals were detected using a microplate reader with excitation at 490 nm and emission at 530 nm. The killing ratio was calculated using the following formula: killing ratio = [(co-culture lysis – spontaneous lysis)/ (maximum lysis – spontaneous lysis)] × 100%.

### In vivo murine tumor experiments

Tumor models were established in female C-NKG mice via intravenous injection of 1.5 × 10⁶ LILRB4 + MM1.S-luc cells. Longitudinal tumor growth was monitored using bioluminescence imaging (Clinx, China) on day 5 post-injection. Mice with successfully established tumors were randomly assigned to four treatment groups. PBS-treated control, NK cell alone, NK cell combined with BiKE, and NK cell combined with BiKE and SR-717. Expanded NK cells were injected intravenously on designated days, followed by intravenous BiKE treatment (1 mg/kg) once daily for five consecutive days, or intraperitoneal SR-717 treatment (30 mg/kg) administered every other day for a total of three doses. The control group was injected with an equal volume of PBS. Prior to bioluminescence imaging for tumor growth assessment, mice were anesthetized with isoflurane to minimize pain and distress. Additionally, mice that cannot eat or drink, or those that have lost more than 20% of their body weight, will be euthanized by cervical dislocation after isoflurane anesthesia, before the experiment concludes.

### Bioinformatics analysis

The datasets for MM analysis were obtained from GSE223060. Survival data were predicted using the TCGA dataset. Publicly available single-cell RNA-seq data were used to classify myeloma cells into seven Clusters through Seurat integration and UMAP dimensionality reduction. The FindAllMarkers function was used to identify subset-specific marker genes, and differential genes with P < 0.01 were analyzed for metabolic enrichment using the scMetabolism package. The CellChat package, along with human cell communication data and the CellChatDB.human library, was used to construct cell communication networks involving ligand-receptors specifically expressed by these subsets. The ‘ggplot2’ package was used to generate volcano plots and heatmaps.

### Statistical analysis

Statistical analysis was conducted using GraphPad Prism 9.0 software. All quantitative data are presented as mean ± standard deviation. One-way comparisons of multiple groups were analyzed using one-way ANOVA, and comparisons of two independent variables were analyzed using two-way ANOVA. Additionally, Brown-Forsythe and Welch ANOVA tests were applied for correction analysis. All experiments were conducted with at least three biological replicates, and differences were considered statistically significant at P < 0.05.

## Results

### Evaluation of LILRB4 expression in MM

**Fig 1:**
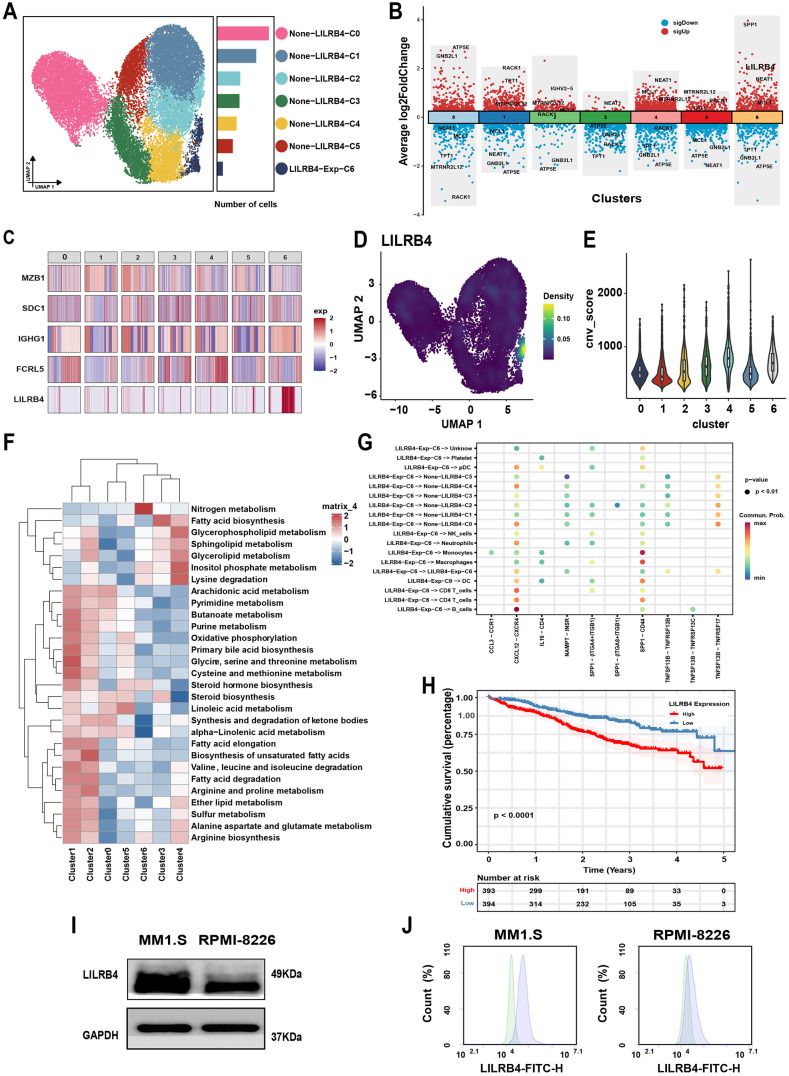
Evaluation of LILRB4 expression in MM. (A) Uniform manifold approximation and projection (UMAP) dimensionality reduction clustering identifies multiple cell subpopulations of multiple myeloma cells. (B) The Volcano plots display the gene expression profiles of each tumor cell subpopulation with significant differences compared to other subpopulations. Log2 (fold change) > 1 and adjusted P < 0.05 were considered as significantly differentially expressed genes. (C) Heatmap showing precise differences in LILRB4 expression across tumor cell subsets. (D) Density plot of LILRB4 expression in tumor cells from UMAP. Color intensity indicates the expression level. (E) Copy Number Variation Score for Tumor Subpopulation Cells. (F) Metabolic pathway enrichment analysis of differentially expressed genes in tumor cell subsets, where color intensity reflects the degree of correlation. (G) Dotplot diagrams display the expression of ligand–receptor pairs between LILRB4^+^MM cells (the initiating point of communication) and other cells. (H) LILRB4-related survival analysis in MM was performed using the TCGA database, with P values calculated by the log-rank test. (I) Western blot analysis was used to detect LILRB4 protein expression levels in MM1.S and RPMI-8226 cells. (J) Flow cytometry was employed to assess the expression of LILRB4 protein in MM1.S and RPMI-8226 cells.

In order to clarify the expression of LILRB4 in primary tumor cells, we first used a single-cell dataset to analyze the expression of LILRB4 in tumor subpopulations, and performed metabolic and cell communication analysis through differential genes. Subsequently, survival analysis related to LILRB4 was conducted using the TCGA database. We performed dimensionality reduction and clustering analysis on the Gene Expression Omnibus (GEO) dataset GSE223060, identifying seven distinct MM clusters, labeled 0–6. Among these, Cluster 6 exhibited significant upregulation of LILRB4 (Fig 1A, B), whereas minimal expression was observed in the other MM tumor cell subsets (Fig 1C, D). Next, copy number variation (CNV) analysis of these tumor cells revealed that Cluster 6 also had higher cellular CNV scores (Fig 1E). Metabolic pathway enrichment analysis of differentially expressed genes in these tumor cell subsets showed a strong association of LILRB4^+^ MM cells with nitrogen metabolism (Fig 1F), this may be associated with high gamma globulin synthesis in MM. MM cells are derived from terminally differentiated B cells [30], cell communication analysis further revealed that this population of cells interacts with B cells via the CXCL12/CXCR4 pathway, LILRB4-positive MM cells are predicted to have some feedback regulation of B cells. Additionally, LILRB4 ⁺ MM cells communicate with monocytes through the SPP1/CD44 axis, which likely contributes to the regulation of the monocyte–macrophage system within the tumor microenvironment (Fig 1G). These data analyses suggest that LILRB4 ⁺ MM cells may play a critical role in tumor progression. Analysis of the TCGA database indicated that elevated LILRB4 expression was linked to shorter disease-free survival (Fig 1H). In order to screen MM cell lines with high expression of LILRB4 to validate the function of BiKE, we investigated the literature and selected MM1. S and RPMI8226 cell lines, LILRB4 expression levels in the MM1.S, and RPMI-8226 cell lines were then evaluated by Western blot. LILRB4 was highly expressed in the MM1.S cell line (Fig 1I), and the flow cytometry results were consistent with these observations (Fig 1J). These findings suggest that LILRB4 may be a potential target for treating MM cells with high malignancy.

### Expression and activity verification of BiKE

**Fig 2:**
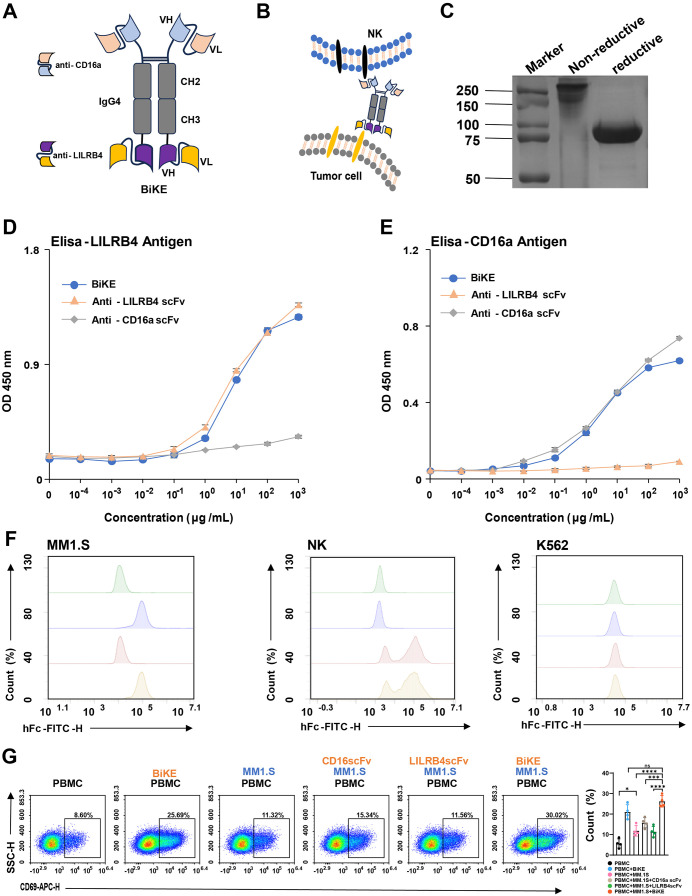
BiKE expression and activity validation. (A) Schematic representation of the BiKE structure. (B) Schematic representation of BiKE-mediated NK cells killing of tumors. (C) BiKE SDS-PAGE of non-reduced and reduced proteins, stained with Coomassie blue. (D) ELISA test measuring the affinity of different concentrations of BiKE to the LILRB4 antigen. (E) Elisa tests the affinity of different concentrations of BiKE to the CD16A antigen. (F) Indirect labeling Flow cytometry was used to assess the binding ability of BiKE to MM1.S, NK, and K562 cells. Goat anti human IgG (H + L) antibody labeled with FITC as the secondary antibody. (G) PBMCs were co-cultured with LILRB4^+^ MM1.S cells for 4 hours. The regulation of CD69 expression in NK cells by monoclonal antibodies and BiKE in the co-culture system was analyzed by flow cytometry. Data are presented as mean ± SD, *P < 0.05, ***P < 0.001, ****P < 0.0001, n = 4 compared to each other group by one-way ANOVA.

To obtain bispecific antibodies (Fig 2A, B), we constructed pVAX expression vectors encoding scFv sequences of anti-LILRB4 and anti-CD16A, each conjugated to the human fragment crystallizable (Fc) domain (hFc). CHO cells were used for antibody expression, and the purified antibody was analyzed by both reduced and non-reduced SDS-PAGE. After reduction, the antibody size was approximately 80 kDa, which was consistent with the expected size (Fig 2C). To assess the affinity of BiKE for the antigen, we simultaneously constructed monoclonal antibodies targeting CD16A and LILRB4. Antigen coating was followed by ELISA to measure the antibody’s affinity (Fig 2D, E). The affinity of BiKE was similar to that of the monoclonal antibody, and its structure did not significantly affect BiKE activity. To further investigate the binding of BiKE to target cells, we employed indirect flow cytometry and found no significant difference in the binding activity of BiKE to antigen targets on MM1.S and NK cells compared to monoclonal antibodies. None of the antibodies bound to K562 cells, which lack the target (Fig 2F). To verify whether BiKE enhances NK cell activity in mediating tumor cell killing, we co-cultured human PBMCs with LILRB4^+^ MM1.S cells. The BiKE group showed the highest upregulation of CD69 on NK cells compared to monoclonal antibodies and other control groups (Fig 2G), indicating the strongest NK cell activation.

### BiKE promotes MM killing by NK cells

**Fig 3:**
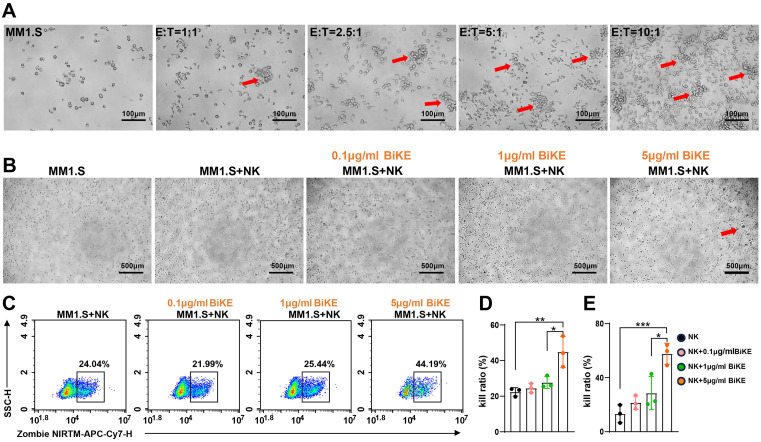
BiKE facilitates the lysis of MM cells by natural killer (NK) cells. (A) Flow cytometry was employed to assess the expression of CD56, CD3, and CD16 in purified and expanded NK cells. (A) The cytotoxic effect of NK cells on MM1.S cells was assessed when co-cultured with varying effector-to-target ratios of MM1.S cells. (B) NK cells were co-cultured 1:1 with MM1.S cells and photographed in bright field when treated with different concentrations of BiKE for 4 hours. (C, D) The effect of various concentrations of BiKE on mediating NK cell-mediated tumor cell killing was evaluated by flow cytometry when NK and MM1.S cells were co-cultured at a 1:1 ratio. (E) MM1.S cells were labeled with calcein and co-cultured with NK cells at a 1:1 ratio for 4 hours. The tumor cell killing ratio was determined based on the calcein release assay. Data are presented as mean ± SD, *P < 0.05, **P < 0.005, ***P < 0.001, n = 3 compared to each other group by one-way ANOVA.

To evaluate the function of BiKE in mediating tumor cell killing by NK cells, PBMCs were isolated from healthy donors and NK cells were sorted using CD56 and CD3 magnetic bead antibodies. After in vitro activation and expansion, more than 95% of the expanded NK cells were CD16-positive (S1 Fig). To avoid masking BiKE function due to an excessively high effector-to-target ratio, we screened for an optimal ratio and selected 1:1 for the subsequent experiments (Fig 3A). NK cells were co-cultured with MM1.S cells at a 1:1 ratio and treated with different concentrations of BiKE. Significant aggregation of MM1. S cells was observed in the BiKE treatment group at a concentration of 5 μ g/ml, indicating that NK cells have an active response to tumor cells (Fig 3B). Flow cytometry analysis was performed immediately after live/dead staining, and results demonstrated that the high-concentration BiKE treatment significantly enhanced NK cell-mediated killing of MM1.S cells (Fig 3C), with an average killing rate of approximately 45% (Fig 3D). To further confirm the cytotoxic activity, a calcein-labeled release assay was performed, showing a consistent trend with the flow cytometry results (Fig 3E).

### Combination of cGAS–STING agonists with the BiKE enhances NK cell–mediated cytotoxicity against MM tumor cells

**Fig 4:**
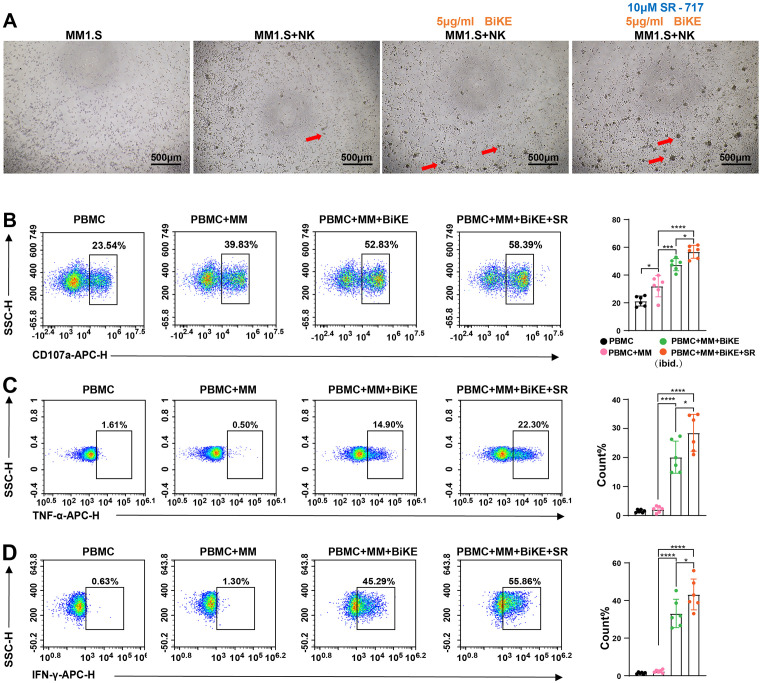
STING Agonists Promote BiKE-Mediated NK Cell Tumor Killing. (A) NK cells were co-cultured with MM1.S cells at a 1:1 ratio, and the combination of SR-717 and BiKE regulated NK cells to effectively kill tumor cells, resulting in a substantial number of tumor agg regates being destroyed. (B) PBMCs were co-cultured with MM1.S cells at a 1:1 ratio to assess the expression of CD107a on NK cells, which were regulated under different conditions of SR-717 or BiKE. (C)Expression level of TNF-α. (D) Expression level of IFN-γ. The experiment was repeated with three different donors. Data are presented as mean ± SD, *P < 0.05, ***P < 0.001, ****P < 0.0001, n = 6 compared to each other group by one-way ANOVA.

The cGAS-STING signaling pathway is integral to innate immune responses, promoting type I interferon secretion, which recruits and activates immune cells to target tumors [31,32]. To harness this immune pathway for enhancing NK cell anti-tumor functions, we applied the STING agonist SR-717 concurrently with BiKE-mediated NK cell killing of tumor cells. Compared with other groups, a large number of tumor cell clusters were observed in the SR-717 combined with BiKE application group. (Fig 4A). In order to better simulate the immune microenvironment in vivo, we co cultured unstimulated PBMCs with MM1. S cells and evaluated the expression of NK cell killing function indicators CD107a (Fig 4B), TNF – α (Fig 4C), and IFN – γ (Fig 4D). We found that in the presence of BiKE, the proportion of CD107a, TNF – α, and IFN – γ positive cells was significantly upregulated, and the addition of SR-717 further enhanced the proportion of positive cells. It can be seen that the combination of SR-717 and BiKE further enhances the killing function of NK cells. To confirm the role of endogenous STING signaling activation in NK cells, we co cultured PBMCs with MM1. S and sorted NK cells using magnetic bead antibodies. We found that the transcription levels of IFNB1 and CCL5 mRNA in NK cells were significantly upregulated in the SR-717 and BiKE combined killing group (S2 Fig). It can be seen that SR-717 further activates NK cells in the presence of BiKE and provides a molecular basis for immune cell recruitment.

### BiKE combined with SR-717 promotes NK cell suppression of tumors in vivo

**Fig 5:**
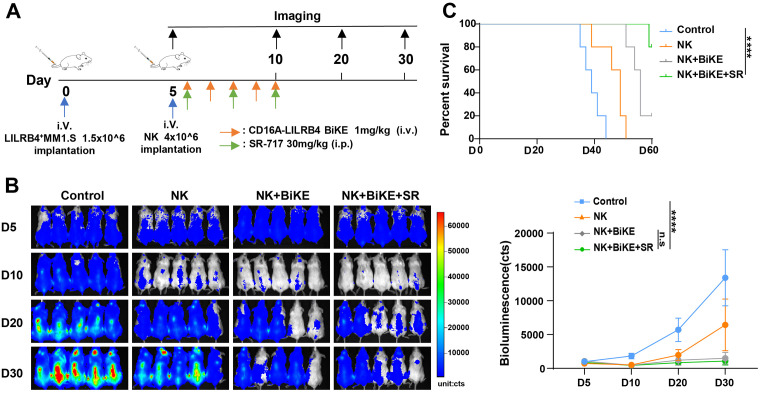
BiKE combined with SR-717 promotes NK cell suppression of tumors in vivo. (A) Schematic of the protocol for NK cell combined antibody treatment of tumors in NKG mice. (B) Representative bioluminescent images, tumor cells after different treatments over time, and consistent quantification of tumor flux using Clinx IVScopeEQ Capture software. Data are presented as mean ± SD, ****P < 0.0001, n = 5 compared to each other group by two-way ANOVA. **(C)** Comparative analysis of overall survival in tumor-bearing mice using the log-rank statistical test.

To assess the effect of bispecific antibodies in mediating tumor suppression by NK cells in vivo, luciferase-expressing LILRB4 + MM1.S cells were expanded in vitro and injected intravenously into NKG mice to establish a disseminated xenograft model (Fig 5A). NK cells from the peripheral blood of healthy donors were activated and expanded in vitro, and their tumor-killing efficacy in vivo was assessed by intravenous injection. On day 5 post-tumor inoculation, tumor growth in the mice was monitored by in vivo imaging. Treatment was initiated once successful xenograft model construction was confirmed. Each mouse received an intravenous injection of 4 × 10⁶ NK cells. The antibody treatment group was administered 1 mg/kg of the bispecific antibody intravenously for 5 consecutive days, while SR-717 was given intraperitoneally at 30 mg/kg every other day for three doses. Tumor progression was continuously monitored by in vivo imaging (Fig 5B). A significant decrease of tumor signal was observed on day 10, whereas the control group exhibited rapid tumor proliferation. By day 20, tumor signals in all groups increased, indicating tumor growth. However, the antibody treatment group demonstrated a significantly slower tumor growth rate compared to the NK cell-only treatment group. Survival analysis was performed on day 60, at which point all mouse experiments were terminated. Statistical analysis revealed that the combination of SR-717 and BiKE significantly prolonged survival in mice treated with NK cells, as compared to other groups (Fig 5C).

## Discussion

Natural killer (NK) cells, a subset of innate lymphoid cells, exhibit potent cytolytic activity, serving as key players in host defense against microbial infections and tumors. Their antitumor effects, coupled with minimal toxicity, make NK cells highly promising effector cells for cancer treatment [33,34]. Blood NK cell counts are positively correlated with a lower cancer risk, while higher NK cell infiltration in tumor tissue correlates with improved treatment outcomes [35]. Donor-derived NK cells are essential in protecting against relapse after hematopoietic cell transplantation (HCT), as they are among the first to reconstitute the recipient’s immune repertoire [36].However, the specialized immunosuppressive microenvironment in MM patients results in decreased NK and NKT cell numbers and impaired effector function, particularly in advanced stages [37–39]. So, direct treatment with bispecific antibodies may not be effective. adoptive NK cell immunotherapy is a good choice. Due to the natural immune characteristics of NK cells, viral transduction efficiency is low, and the preparation and application costs of CAR-NK cells are high [40]. Therefore, combining bispecific antibodies with NK cell therapy represents an effective strategy to fully exploit the innate advantages of NK cells.

LILRB4 is recognized as an immune checkpoint on bone marrow cells [41]. LILRB4 is highly expressed during the early stages of plasma cell malignancy in MM (CD38 + CD138 + CD19 + CD56 +), and is considered a marker of aggressive proliferation in MM. Relapsed or refractory multiple myeloma (RRMM) expresses higher levels of LILRB4 than newly diagnosed multiple myeloma (NDMM). Furthermore, LILRB4+ MM induces the increased production of monocyte-myeloid derived suppressor cells (M-MDSC) and suppresses T-cell infiltration [14]. Our findings support this, showing that LILRB4+ MM cells are strongly associated with nitrogen metabolism and may contribute to aberrant immunoglobulin synthesis in MM. Furthermore, we observed that LILRB4+ MM cells exhibit strong cellular communication with B cells and monocytes, which may align with the regulation of early plasma cell carcinogenesis by LILRB4+ MM and M-MDSC production as reported by Gong et al [14]. Additionally, despite its low expression on dendritic cells, monocytes, macrophages, progenitor mast cells, endothelial cells, and osteoclasts [42], LILRB4 does not pose a graft-versus-host disease risk due to the unique recognition and killing pattern of NK cells. Moreover, it has been demonstrated that LILRB4 blockade does not significantly interfere with normal hematopoiesis [13,43]. Thus, this target exhibits reliable safety.

To target and kill this group of cells, we report the preclinical development of a new antibody-based NK cell engager, BiKE: LILRB4/CD16A, which targets LILRB4 on malignant cells and co-engages CD16A on NK cells. It directly signals through CD16 to activate NK cells [43]. CD16A on NK cells binds to antibody-coated cells, triggering antibody-dependent cell-mediated cytotoxicity. Our IgG-like bispecific antibodies, with a longer half-life than small-molecule antibodies lacking the Fc segment, do not significantly cross the blood-brain barrier [44]. BiKE operates through a mechanism similar to Fc-mediated molecules, triggering ADCC and ADCP, and is unaffected by CD16A polymorphisms [12]. It significantly activates NK cells and enhances the expression of cytotoxic molecules, such as CD107a, TNF-α, and IFN-γ, in vitro. It inhibits the growth of target tumor cells in vivo and prolongs the survival of tumor-bearing mice. Furthermore, activated NK cells are not eliminated by ADCC because the IgG4 segment has a high affinity for FcγRI but a weak affinity for other FcγRs, resulting in low Fc-mediated effector activity [45].

To further enhance the efficacy of immunotherapy, we utilized cGAS-STING agonists combined with BiKE therapy. The cGAS-STING pathway is a critical component of innate immune responses against cancer. Studies have demonstrated that cancer cells with downregulated cGAS-STING activity are able to evade immune surveillance [46]. In this study, we introduced SR-717 into the complete killing system and demonstrated a significant enhancement of NK cell killing activity in vitro. Previous studies have shown that the addition of exogenous cGAS-STING agonists triggers STING activation and IFN-β production in myeloid and B cells, rather than directly activating NK cells, which are downstream of interferons. IFN-β then activates NK cells through direct or indirect pathways [47]. Upon activation, NK cells secrete IFN-β and CCL5, which further recruit additional immune cells to the tumor site. However, our in vivo experiments had limitations: the immunodeficient mice lacked T, B, and NK cells, and macrophage and dendritic cell functions were impaired. Additionally, the systemic application of SR-717 may not be sufficient to fully activate NK cells in this in vivo model, which could explain the suboptimal results observed in the BiKE and SR-717 combination group.

LILRB4 is expressed in a subset of highly aggressive multiple myeloma (MM) variants and may not be sufficient to rapidly resolve the disease in clinical therapy. To enhance the clinical applicability of antibodies, it may be necessary to develop trispecific antibodies targeting widely expressed markers, such as BCMA and GPRC5D, in the future. Furthermore, humanization of antibody sequences is a key optimization step in future antibody development, aimed at reducing immunogenicity and minimizing side effects. Simultaneously, preclinical animal models must also be optimized, as the molecular mechanism by which cGAS-STING combined with bispecific antibodies mediates NK cell killing of tumor cells can be more accurately explored in humanized animal models with a functional human immune system.

## Supporting information

S1 Fig In vitro expansion and culture of NK cells. After isolating human peripheral blood NK cells, they were expanded and cultured in vitro for 14 days. Subsequently, NK cell purity and CD16 expression were assessed.
(TIFF)

S2 Fig The mRNA expression of IFNB1 and CCL5 in NK cells after co culture of PBMC and MM1. S cells. Co-culture PBMCs with MM1.S cells and treat with BiKE or BiKE combined with SR-717 for 4 hours. Subsequently, NK cells are sorted using magnetic bead antibodies, and RNA is extracted. RT-qPCR is performed to assess the mRNA expression of IFNB1 and CCL5. Data are presented as mean ± SD, *P < 0.05, **P < 0.005, n = 3 compared by *t* test.
(TIFF)

S1 Data Raw data. (ZIP)
