# Supplementary figures and images for: The combination of LILRB4-targeting NK cell engagers and cGAS–STING agonists enhances the anti–multiple myeloma immune activity of NK cells
Source: PLoS One. 2025 Dec 19;20(12):e0339375. doi: 10.1371/journal.pone.0339375 (PMC12716741; doi:10.1371/journal.pone.0339375)

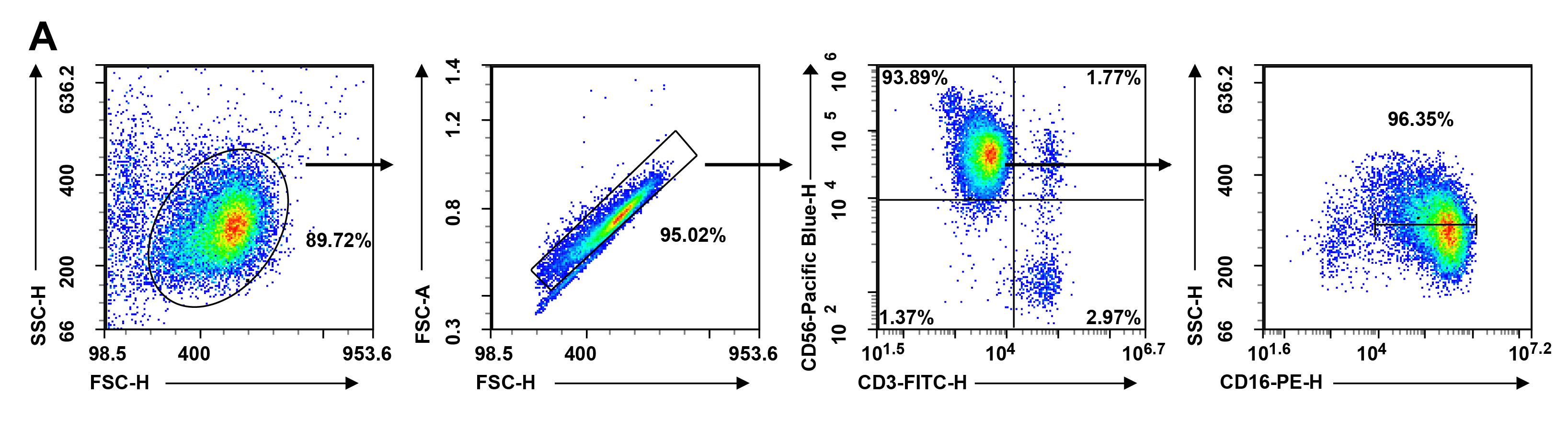

Supplement: S1 Fig — After isolating human peripheral blood NK cells, they were expanded and cultured in vitro for 14 days. Subsequently, NK cell purity and CD16 expression were assessed. (TIFF) [file pone.0339375.s001.tiff]

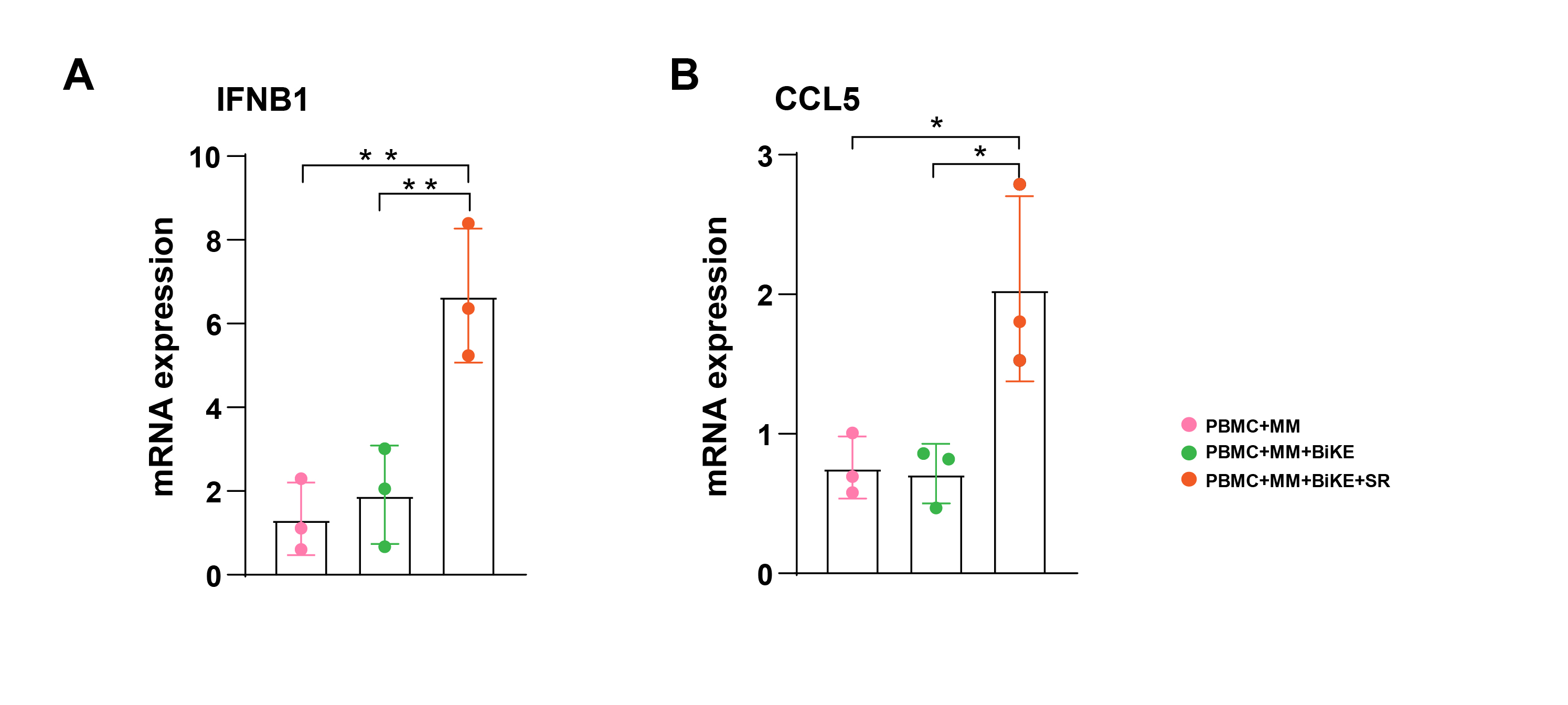

Supplement: S2 Fig — S cells. Co-culture PBMCs with MM1.S cells and treat with BiKE or BiKE combined with SR-717 for 4 hours. Subsequently, NK cells are sorted using magnetic bead antibodies, and RNA is extracted. RT-qPCR is performed to assess the mRNA expression of IFNB1 and CCL5. Data are presented as mean ± SD, *P < 0.05, **P < 0.005, n = 3 compared by t test. (TIFF) [file pone.0339375.s002.tiff]

**Fig 3A**

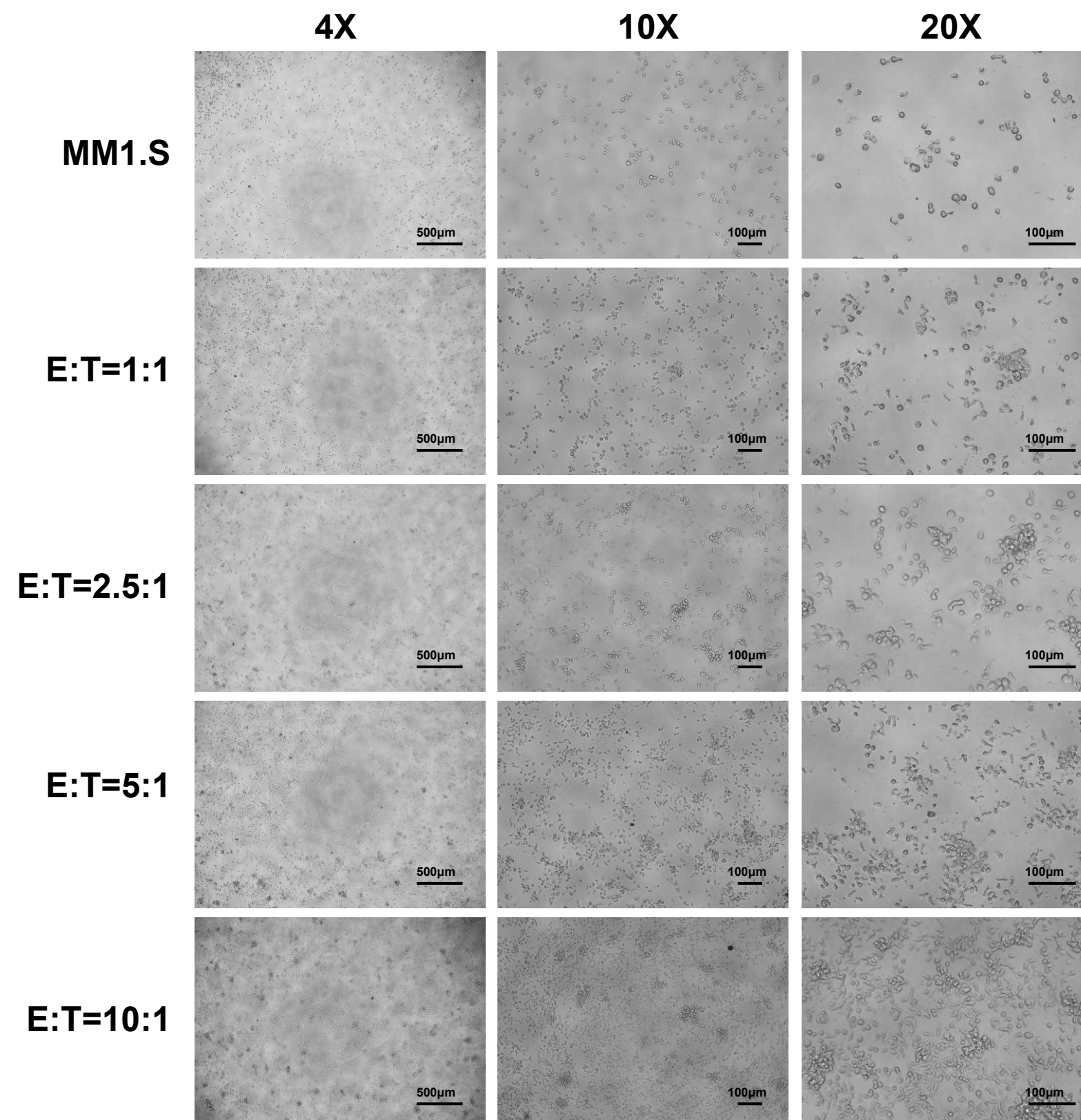

**Co culture of NK cells and MM1. S cells**

Supplement: S1 Data — (ZIP) [file pone.0339375.s003.zip › Raw data/Fig 3A.pdf]

**Fig 3B**

**MM1.S**

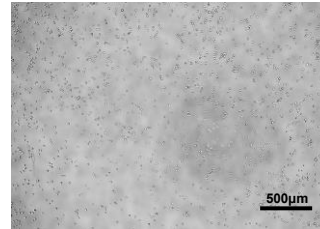

**MM1.S+NK**

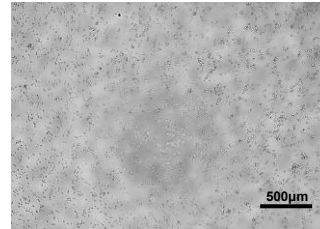

**MM1.S+NK  
0.1 μg/ml BiKE**

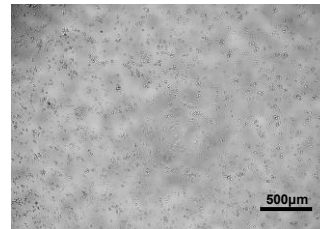

**MM1.S+NK  
1 μg/ml BiKE**

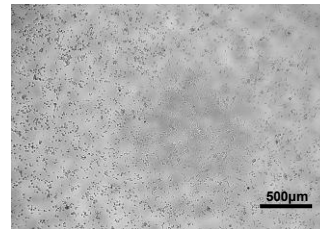

**MM1.S+NK  
5 μg/ml BiKE**

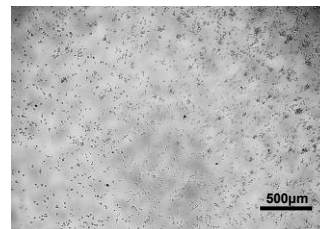

Supplement: S1 Data — (ZIP) [file pone.0339375.s003.zip › Raw data/Fig 3B.pdf]

**MM.1S**

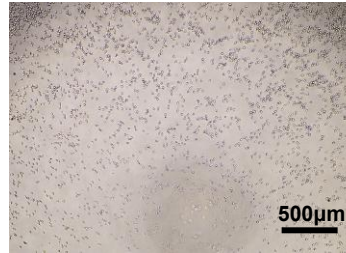

**MM.1S+NK**

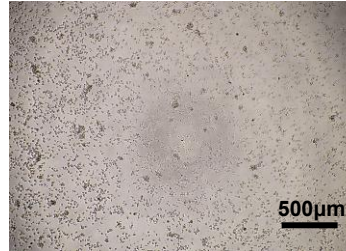

**MM.1S+NK**  
**5 μg/ml BiKE**

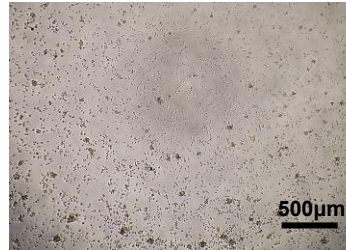

**MM.1S+NK**  
**5 μg/ml BiKE**  
**10 μM SR-717**

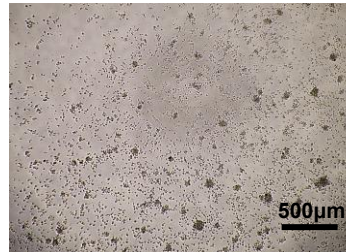

Supplement: S1 Data — (ZIP) [file pone.0339375.s003.zip › Raw data/Fig 4A.pdf]

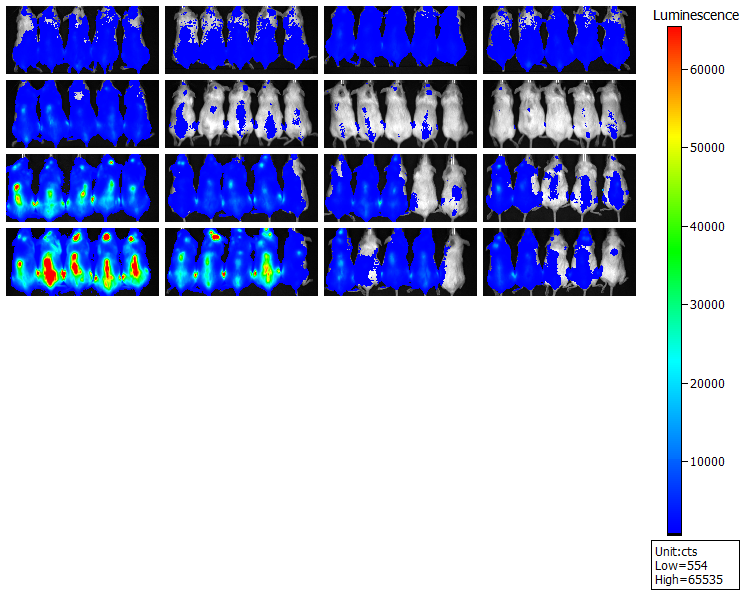

Supplement: S1 Data — (ZIP) [file pone.0339375.s003.zip › Raw data/Fig 5B/Fig 5B.tif]
