# Supplementary material for: The combination of LILRB4-targeting NK cell engagers and cGAS–STING agonists enhances the anti–multiple myeloma immune activity of NK cells
Source: PLoS One. 2025 Dec 19;20(12):e0339375. doi: 10.1371/journal.pone.0339375 (PMC12716741; doi:10.1371/journal.pone.0339375)
Supplement: S1 Data — (ZIP) [file pone.0339375.s003.zip › Raw data/S1_raw_images.pdf]

Figure 1I

GAPDH

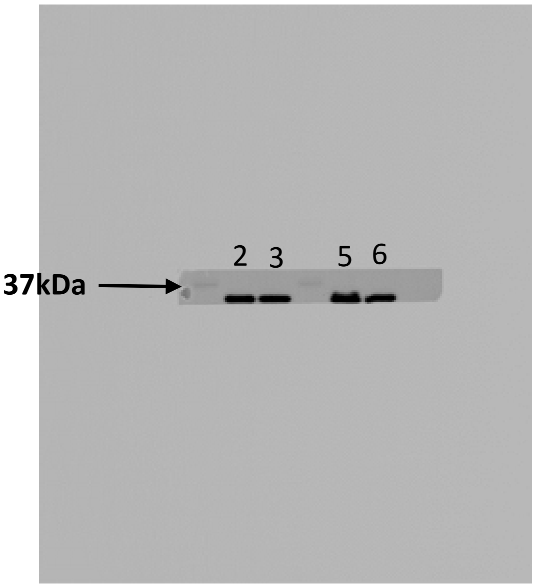

Lane2	MM1.S
Lane3	RPMI-8226
Lane5	MM1.S
Lane6	RPMI-8226
Lanes 5 and 6 are duplicates	

LILRB4

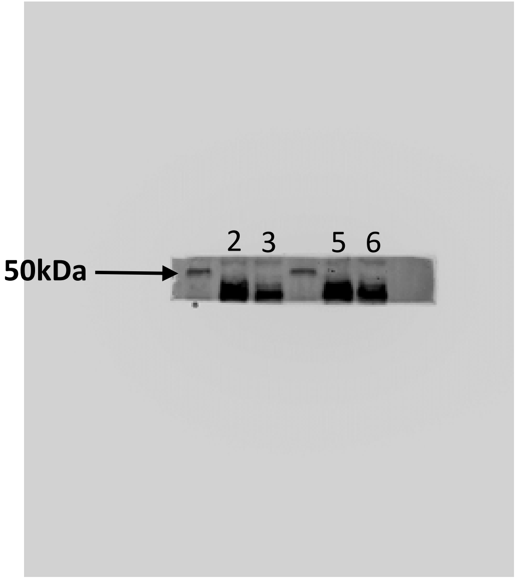

Lane2	MM1.S
Lane3	RPMI-8226
Lane5	MM1.S
Lane6	RPMI-8226
Lanes 5 and 6 are duplicates	

Figure 2C

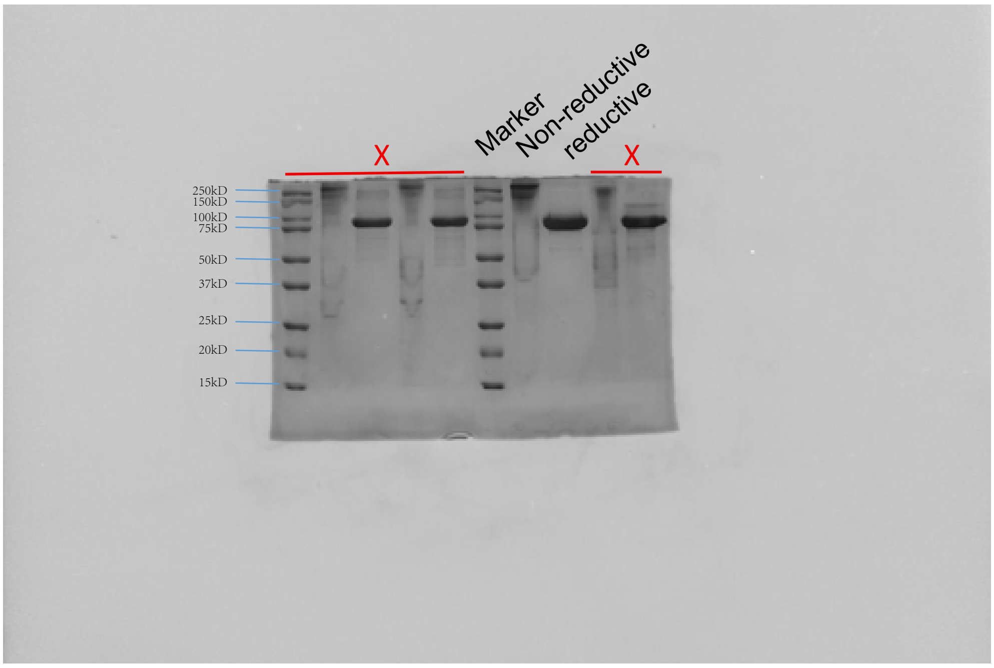
X
Marker
Non-reductive
reductive
X
250kD
150kD
100kD
75kD
50kD
37kD
25kD
20kD
15kD
